# Development and Evaluation of a Novel Self-Etch Dental Adhesive Incorporating Graphene Oxide–Zirconia (GO-ZrO_2_) and Hydroxyapatite–Zinc (HA-Zn) for Enhanced Bond Strength, Biocompatibility, and Long-Term Stability

**DOI:** 10.3390/nano15110803

**Published:** 2025-05-27

**Authors:** Norbert Erich Serfözö, Marioara Moldovan, Doina Prodan, Nicoleta Ilie

**Affiliations:** 1 Department of Conservative Dentistry and Periodontology, University Hospital, Ludwig-Maximilians-University, LMU, 80333 Munich, Germany; 2 Institute of Chemistry Raluca Ripan, Babes-Bolyai University, 737322 Cluj-Napoca, Romania

**Keywords:** graphene, hydroxyapatite, self-etch dentin adhesive, bond strength, cytotoxicity

## Abstract

The aim of this study was to develop an experimental self-etch dental adhesive (SE) by synthesizing graphene oxide–functionalized zirconia (GO-ZrO_2_) and hydroxyapatite–functionalized zinc (HA-Zn) as inorganic powders together with bis-GMA _(0–2)_ (bisphenol A-glycidyl methacrylate) oligomers as main components of the organic matrix. The adhesive was compared to the current gold standard adhesive Clearfill SE Bond 2 (CSE) using cytotoxicity assays, shear bond strength (SBS) tests, and resin–dentin interface analyses. Cytotoxicity assays with human gingival fibroblasts (HGF-1) revealed reduced cell viability at early time points but indicated favourable biocompatibility and potential cell proliferation at later stages. SBS values for the experimental adhesive were comparable to CSE after 24 h of storage while aging did not significantly affect its bond strength. However, SBS exhibited more consistent resin tag formation and higher Weibull modulus values post-aging. A scanning electron microscopy (SEM) analysis highlighted differences in resin tag formation, suggesting the experimental adhesive relies more on chemical bonding than micromechanical interaction. The experimental adhesive demonstrated promising potential clinical properties and bond durability due to the integration of GO-ZrO_2_ and HA-Zn fillers into the adhesive.

## 1. Introduction

More than one-third of the global population lives with untreated dental caries. This disease is one of the most widespread non-communicable diseases and a significant public health issue, affecting over 2 billion people worldwide [1].

The modern treatment of dental caries is achieved by direct resin-based composite (RBC) restorations. This is the primary procedure in dental practices worldwide due to its ability to preserve sound tooth structure and its superior aesthetic appearance. Its applicability depends on the usage of resin-based dentin bonding agents (DBAs) to bond to the tooth structure (enamel or dentin) [2].

The extensive clinical use of DBAs has driven continuous modifications in their composition and formulation, leading to the development of successive generations of dental adhesives. In line with a trend toward simplification and a more user-friendly approach, self-etch adhesives were developed to reduce application steps, shorten application time, and lower post-operative sensitivity as compared to etch-and-rinse adhesives [3]. Unlike etch-and-rinse systems, self-etch adhesives incorporate acidic monomers that simultaneously condition and prime the dental substrate eliminating the need for a separate etching step [3]. The reduced incidence or complete absence of clinical post-operative sensitivity with self-etch adhesives is due to their less aggressive and more superficial interaction with dentin. Additionally self-etch adhesives provide a substrate for acidic monomers to chemically interact with the dentinal tissue (mild self-etch adhesives) which is believed to enhance bond durability [3,4].

However, regardless of the remarkable progress that has been achieved in the field of adhesive dentistry, recurrent decay is still the main reason for failure of direct RBC restoration [5]. The occurrence of recurrent decay suggests that the bond between the tooth and RBCs is inadequate to endure the physical, chemical, and mechanical challenges of the oral environment as well as the contraction forces generated during polymerization [6,7].

To address these problems, current research focuses on developing innovative strategies such as incorporating novel reinforcement particles into adhesive systems. These particles aim to enhance the bond strength, biocompatibility and durability of the restoration interface, potentially reducing the risk of recurrent decay [8].

One such particle is graphene, which has gained significant attention since Novoselov et al. successfully isolated it from graphite using mechanical exfoliation [9]. Its unique two-dimensional properties and conjugated aromatic structure imparts graphene with exceptional key characteristics which include a large surface area (~2600 m^2^g^−1^), excellent electrical (2.5 × 10^5^ cm^2^ V^−1^ s^−1^) and thermal conductivity (3000–5000 W mK^−1^) [10,11], superior mechanical properties (Young’s modulus ~1 TPa), tensile strength (~130 GPa) [12,13], biocompatibility, and antibacterial properties [14,15].

The versatile properties of graphene and its derivatives graphene oxide (GO) and reduced graphene oxide have shown considerable promise across various dental applications. Recent studies report that RBCs with graphene derivatives provide protection for dentinal tissue against decalcification [16], while functionalization with zirconium oxide (ZrO_2_) has led to improved mechanical properties and material reliability [15]. Additionally, graphene has been beneficial in the development of dental materials for bone regeneration [17], as coatings for dental implants with therapeutic effects for managing peri-implantitis [18] and in the mechanical reinforcement of prosthetic materials based on polymethyl methacrylate [19]. Graphene has also been shown to improve the mechanical properties of experimental resin-based luting materials used in posterior restorations [20].

Graphene and its derivatives have also been successfully incorporated into dental adhesives. AlFawaz et al. demonstrated that an HA-GO filler at concentrations of 0.5% by weight and 2% by weight in an etch-and-rinse dental adhesive resulted in improved bond strength regardless of the aging process [21]. Similarly, Bin-Shuwaish and Maawadh showed that incorporating GO at concentrations of 0.5% by weight and 2% by weight in experimental adhesives significantly improved bond strength (2% group) and increased the degree of conversion (0.5% group) [22].

An advantageous feature of GO lies in the inherent presence of oxygen functional groups (hydroxy, epoxy, and carboxy groups) distributed over its surface and edges, which facilitate the chemical bonding of graphene with other compounds [12]. Among the various options, ZrO_2_ nanoparticles stand out as promising candidates for attachment to the surface of GO due to their exceptional hardness, mechanical strength, chemical and thermal stability, and wear resistance [15,23].

Building on these advancements, hydroxyapatite (HA) nanoparticles have also been integrated into DBAs, where their bioactivity, biocompatibility, and remineralisation properties contribute to improved bond strength and reduced hybrid layer degradation [24]. Additionally, zinc oxide (ZnO) nanoparticles have been used in adhesive formulations, demonstrating multifunctional benefits such as enhanced antimicrobial activity [25], mechanical reinforcement, and enhanced adhesive durability [25,26].

Together, these advancements underline the potential of the nanoparticles described above and motivate the development of an experimental self-etch adhesive (SE) formulation with zirconia functionalized with graphene oxide (GO-ZrO_2_) and zinc functionalized with hydroxyapatite (HA-Zn). This innovative approach aims to enhance the biocompatibility and bond strength of the adhesive system to dentin.

We hypothesized that the experimental adhesives (SEs) would behave similarly to the gold standard adhesive Clearfill SE Bond 2 (CSE) in terms of: (a) bond strength and interaction with dentin; (b) behaviour during aging; and (c) cellular toxicity up to 3 months.

## 2. Materials and Methods

An experimental self-etch dental adhesive system (SE) was developed by synthesising novel bis-GMA _(0–2)_ oligomers and fillers. The formulation was based on zirconia functionalized with graphene oxide and zinc functionalized with hydroxyapatite. The adhesive system (SE) was developed as a two-component system and consisted of a primer for mild conditioning and infiltration of the dentin and a bonding agent to promote adhesion between the conditioned dentin and the resin-based composite. Comparative analyses were conducted using the established gold standard self-etch dental adhesive (Clearfill SE BOND 2, Kuraray Co., Ltd., Kurashiki, Japan, abbreviation CSE). A low-shrinking bulk fill resin-based composite (Admira Fusion X-tra, Voco GmbH, Cuxhaven, Germany, abbreviation AF) was used with all adhesives. The evaluation included the assessment of cytotoxicity towards human gingival fibroblasts (HGF-1), bond strength to dentin under conditions of thermal aging (thermal cycling, TC), and a detailed examination of the resin–dentin interface morphology. The aim was to comprehensively characterise the performance, biocompatibility, and structural integrity of the experimental adhesive in comparison to existing standards.

### 2.1. Synthesis of the Experimental Self-Etch Adhesive

The chemical composition of the experimental dental adhesive is presented in Table 1. The organic matrix consisted primarily of 2,2-bis[p-(2′-hydroxy-3′-methacryloxypropoxy)phenyl]-propane—bis-GMA _(0–2)_. For the synthesis of the upper bis-GMA _(0–2)_ oligomers, a low molecular weight epoxy resin, namely, the “Dinox 010S” resin, was used. It consisted of 84% monomer (diglycidyl bisphenol A ether), 15% dimer, and 1% trimer (% mole). Prior to the reaction, the catalyst TPP (triphenylphosphine, Merck KGaA, Darmstadt, Germany) and the inhibitor BHT (2,6-di-tert-butyl-4-methylphenol, Merck) were dissolved in the methacrylic acid. The resin “Dinox 010S” reacted with methacrylic acid at a temperature of 60 °C and in the absence of solvents, whereby each oligomer of the epoxy resin RE_n_ (n = 0, 1, 2) was converted into an oligomer bis-GMA_n_ (n = 0, 1, 2), the first bis-GMA_n_ oligomer being considered as the bis-GMA monomer. The synthesis was conducted in a glass reactor equipped with a mechanical stirrer, condenser, thermometer, and flanged cap, maintained at a constant temperature of 60 °C and continuously stirred for 34 h. The completion of the reaction was confirmed by titration against 0.1 N NaOH, using acetone as the solvent and bromthymol blue as the indicator, until the residual methacrylic acid was reduced below 0.5%.

**Figure 1:**
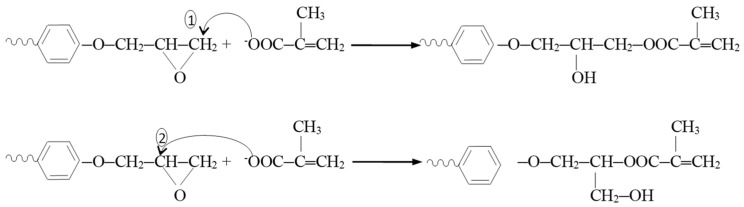
Reaction mechanism in the synthesis of the bis-GMA _(0–2)_ oligomers.

The reaction mechanism in the synthesis of the bis-GMA _(0–2)_ oligomers is depicted in Figure 1 and involves the opening of the epoxy cycle by attacking the nucleophilic reactant, the methacryloyloxy ion, at the C1 atom, so that at the end of the molecule, a linear (“normal”) structure is formed. However, the attack on the C2 atom is possible, forming in this case a branched structure (“isomeric”). As a result, three isomers may occur as a result of the synthesis reaction:

1. n, n: isomer with “normal” terminal groups at both ends of the molecule.
2. n, i: the isomer with a “normal” terminal group at one end and an “isomer” group at the other end of the molecule.
3. i, i: the isomer with “isomeric” terminal groups at both ends of the molecule.

Following synthesis, the bis-GMA _(0–2)_ oligomer mixture was combined with dilution monomers, TEGDMA (triethylene glycol dimethacrylate) and HEMA (2-hydroxyethyl methacrylate), forming the organic matrix. Polyacrylic acid (PAA) was added to adjust the acidity of the mixture. Overall, the monomer mixture constituted 97% by weight of the adhesive formulation, while additives—including polymerization initiators, accelerators, inhibitors, photosensitizers, antioxidants, and UV stabilizers—comprised the remaining 3%. The photoinitiation system specifically consisted of CQ (camphorquinone, 0.5% by weight relative to the liquid mixture) as the photosensitizer, and polymerization accelerators DMAEMA (2-(dimethylamino)ethyl methacrylate) and EDAB (ethyl-4-dimethylaminobenzoate), acting as a co-accelerator to facilitate polymerization, collectively at 1% relative to the total monomer mass. BHT additionally functioned as a UV stabilizer.

Finally, inorganic fillers synthesized specifically for this research were incorporated into the monomer mixture at a concentration of 7% by weight. This incorporation was performed under reduced lighting conditions to prevent premature polymerization, using a T18 disperser (IKA^®^-Werke GmbH & Co., Ltd., KG, Staufen, Germany) operating at 700 rpm for 15 min.

#### 2.1.1. GO-ZrO_2_ Filler Synthesis

GO was synthesized from natural graphite using a modified Hummer’s method [27]. To prepare the GO suspension, 75 mg of GO was added to 10 mL of double-distilled water and sonicated for 15 min to achieve dispersion. Separately, 1.5 g of ZrO_2_ powder (Merck KGaA, Darmstadt, Germany) was dispersed in 15 mL of NaOH solution (pH 9.5) through ultrasonic treatment for 15 min. The two suspensions were then combined, and the mixture was further sonicated for an additional 45 min. The resulting colloidal suspension was stirred magnetically at 50 °C until fully dried, followed by annealing at 300 °C for 15 min under an argon atmosphere.

#### 2.1.2. HA Synthesis

HA was synthesized using wet chemical precipitation methods with variations in raw materials, reaction conditions, and heat treatments. The synthesis began with a 0.5 M calcium hydroxide solution Ca(OH)_2_, prepared via the hydrolysis of calcium oxide (3% CaO mixed with 97% distilled water) and a 0.3 M orthophosphoric acid solution H_3_PO_4_ in stoichiometric proportions. The reaction was carried out at 80 °C for 3 h with the pH maintained at 7.5 by the dropwise addition of 3% ammonia solution. This process yielded a translucent gel as the reaction product matured at room temperature (23 °C) for 70 h.

The primary reaction is represented below:

6 H_3_PO_4_ + 10 Ca(OH)_2_ = Ca_10_(PO_4_)_6_(OH)_2_ + 18 H_2_O

During the maturation process, pH changes were observed and attributed to the hydrolysis of the phosphatic precursor. The hydrolysis products likely formed complexes with calcium ions in solution. Subsequently, the interaction between the hydrolysed phosphorus compounds and calcium ions resulted in the formation of derivatives containing the Ca-O-P base group.

#### 2.1.3. HA-Zn (Hydroxyapatite–Zinc) Preparation 

An aqueous suspension containing 30% ZnO was introduced into the HA gel, and an HA-ZnO nanocrystal suspension was formed. The nanocrystal suspension was kept for maturation for one week at room temperature. The next step was vacuum filtration, followed by rinsing three times with distilled water. The resulting powder was dried at 100 °C for 12 h. A heat treatment was then applied for 2 h at 500 °C

### 2.2. Shear Bond Strength (SBS) Test

Eighty dentin sections were prepared by sectioning freshly extracted human third molars, which had been cleaned of soft tissue and debris and stored in a 0.4% sodium azide solution at 4 °C. Sections were obtained 2 mm below the central groove in a bucco-lingual direction using a water-cooled low-speed diamond saw (Isomet, Buehler, Lake Bluff, IL, USA). The dentin samples were randomly allocated into two groups (n = 40 each). Each dentin section was positioned on a glass surface using double-sided adhesive tape, and a steel cylinder (19 mm height, 19 mm diameter) was centred on top. The cylinder was filled with a methacrylate embedding resin (Technovit 4004, Kulzer, Wasserburg, Germany). To create a standardized smear layer, the dentin surfaces were ground with 600-grit silicon carbide abrasive paper (Hermes, EXAKT Advanced Technologies GmbH, Norderstedt, Germany) using a grinding and polishing machine (Leco Corp., SS-200, St. Joseph M, USA) [28]. The primer was applied to the dentin surface using a microbrush, allowed to remain there for 20 s, and then gently air-dried. The adhesive was then applied for 10 s, followed by gentle air-drying and light curing for 10 s using an LED curing unit (Bluephase Style, Ivoclar-Vivadent, Schaan, Liechtenstein) operating at an irradiance of 1386 mW/cm^2^. The irradiance, radiant exposure, and spectral distribution of the curing unit were measured using a USB4000 spectrometer integrated into the MARC—Resin Calibrator system (Bluelight Analytics Inc., Halifax, NS, Canada). A silicone mould (h = 4 mm, Φ = 3 mm) was positioned on the bonded dentin surface, filled with a bulk-fill resin-based composite (AF) in a single increment, and light-cured for 20 s. Specimens were immersed in distilled water and kept at 37 °C for 24 h in the absence of light. Following storage, half of the samples from each group underwent TC using a dedicated device (Willytec, Dental Research Division, Munich, Germany), consisting of 10,000 alternating exposures to 5 °C and 55 °C, with each temperature maintained for 30 s and a 5 s transfer interval. Shear bond strength was evaluated using a universal testing machine (Zwick Roell 72.5, Ulm, Germany) by applying a load perpendicular to the bonding interface at a constant rate of 0.5 mm/min until failure occurred. Bond strength values, expressed in megapascals (MPa), were obtained by dividing the load at failure (N) by the bonded surface area (mm^2^).

### 2.3. Fractography

To identify the underlying fracture pattern, specimens were examined utilizing stereomicroscopy (Stemi 508, Carl Zeiss AG, Oberkochen, Germany). The fracture pattern was identified and classified in three distinctive categories: adhesive (fracture at the composite–dentin interface), cohesive (fracture in one of the two substrates), and mixed (fracture starts adhesive and continues cohesive in one of the two substrates). All samples exhibited adhesive failure, rendering all data applicable for the SBS test. Samples were subsequently subjected to an analysis using Axio Vision software version 4.8 (Carl Zeiss AG,) to determine the percentage of adhesive left on the dentin surface. The mARI (modified Adhesive Remnant Index) was subsequently established with the following criteria: score 1 (0% to 25% of the adhesive film remaining on the dentin surface), score 2 (25% to 50% of the adhesive film remaining on the dentin surface), score 3 (50% to 75% of the adhesive film remaining on the dentin surface) and score 4 (75% to 100% of the adhesive film remaining on the dentin surface).

### 2.4. Scanning Electron Microscopy (SEM) Analysis

Dentin slices were used to prepare bonded specimens for the SEM analysis, as previously described. Each experimental group included four samples. After adhesive application, AF was applied in a single increment to fully cover the bonded dentin surface. The specimens were then sectioned longitudinally to expose the dentin–adhesive–composite interface and embedded in methacrylate resin using stainless steel cylinders. Specimen surfaces were sequentially finished under water cooling using silicon carbide abrasive papers of increasing grit size (1200, 1500, 2000, and 2400) and polished with diamond suspensions (DP-Spray, Struers GmbH, Puch, Austria) applied to textile pads (DP-Pan, 200 mm, Struers GmbH) in descending particle sizes of 6 µm, 3 µm, and 1 µm. Between each polishing step, samples were ultrasonically cleaned for 5 min in a dedicated cleaning unit (RK 31, Badelin Electronic, Berlin, Germany). Subsequently, the specimens were subjected to demineralization using 6 N hydrochloric acid (Sigma-Aldrich Co., Ltd. St. Louis, MO, USA) and deproteinization in a 12% sodium hydroxide solution (Sigma-Aldrich Co., Ltd.). After thorough rinsing with distilled water and air drying, dehydration was performed using a graded ethanol series (25%, 50%, 75% for 20 min each; 95% for 30 min; and 100% for 60 min). Dried specimens were affixed to aluminium stubs, gold-coated under vacuum, and examined using a field-emission scanning electron microscope (Zeiss Supra 55 VP, Carl Zeiss AG, Germany) at 10 kV. SEM images were taken from three randomly selected areas per specimen at 500× magnification to evaluate the thickness of the adhesive layer and the number of resin tags within the dentinal tubules. For each image, the adhesive layer thickness (in μm) was measured at five randomly chosen locations. The images were divided into 50 μm segments, and the resin tags observed within the dentinal tubules in each segment were counted. The resin tag penetration number (RTPN) was defined as the number of tags per 50 μm. All measurements were performed using the calibrated scale bar in Adobe Photoshop (Version 7.0, Adobe Inc., San Jose, CA, USA).

In addition, bright-field transmission electron microscopy was used to describe the morphology and dispersion of the nanoparticles using a Hitachi H-7650 120 kV Automatic Microscope, Japan.

### 2.5. Cell Culture

HGF-1 (ATCC^®^ CRL-2014™) were grown on sterile cell culture dishes (CellStar^®^, Greiner Bio-One International GmbH, Kremsmünster, Austria) with a nominal size of 100 mm with side heights of 20 mm. Cells were cultured under standard conditions and maintained in a humidified incubator (HERACELL 150i, Thermo Scientific, Waltham, MA, USA) at 37 °C with 5% CO_2_ in a 95% air environment until reaching approximately 80% confluence. Cultivation was performed in high-glucose Dulbecco’s Modified Eagle Medium (DMEM; Sigma-Aldrich Co., Ltd.) supplemented with 10% foetal bovine serum (FBS) and 1% penicillin–streptomycin (PenStrepFa; Sigma-Aldrich Co., Ltd.). Once the desired confluence was achieved, the cells were gently rinsed with Dulbecco’s phosphate-buffered saline (DPBS; Sigma-Aldrich Co., Ltd.) and detached using a trypsin–EDTA solution (0.25% trypsin, 0.53 mM EDTA). Cells were counted using a mix of 10 μL cell suspension and 10 μL trypan blue solution (T8154, Sigma-Aldrich Co., Ltd.) added to a Neubauer improved haemocytometer counting chamber (Paul Marienfeld GmbH & Co., Ltd. KG, Lauda-Königshofen, Germany). The medium was changed 3 times weekly, and cells were monitored using an inverted phase-contrast microscope (Axiovert 40 C, Carl Zeiss AG). The cells were used between the 7th and the 13th passages for the cytotoxicity assays.

### 2.6. Test Specimen and Eluate Preparation

Freshly extracted human third molars were preserved and cleaned according to the procedure previously outlined. Each tooth was sectioned horizontally into 2 mm thick slices using a water-cooled low-speed diamond saw. The surface areas of 21 dentin slices were determined using a stereomicroscope (Stemi 508, Carl Zeiss AG) equipped with a camera attachment and Axio Vision software version 4.8 (Carl Zeiss AG). After surface measurement, the dentin slices were sterilized and evenly assigned to the respective adhesive groups. All bonding procedures were carried out under sterile conditions within a horizontal laminar airflow cabinet (Holten LaminAir, Thermo Scientific, Waltham, MA, USA). Primer was applied to the dentin surface using a sterile microbrush for 20 s and gently air-dried for 5 s. The bonding agent was then applied, air-dried for another 5 s, and light-cured for 20 s. The treated dentin slices were then immersed in 3 mL of complete culture medium in 15 mL conical tubes (Falcon, BD Biosciences, Franklin Lakes, NJ, USA), following ISO 10993-12 guidelines with a surface area-to-volume ratio of 117.8 mm^2^ per ml of medium [29]. Samples were incubated at 37 °C, and eluates were collected in triplicate from each adhesive group and from cell culture medium without specimens (control). Eluate collection occurred at 24 h, 48 h, 72 h, 10 days, 1 month, and 3 months. After each time point, the medium was replaced with 3 mL of fresh complete culture medium. All collected eluates were stored at −20 °C until cytotoxicity testing.

### 2.7. Cytotoxic Assay

To evaluate the potential cytotoxic effects of the materials on the HGF-1 cells, a WST-1 (4-[3-(4-Iodophenyl)-2-(4-nitro-phenyl)-2H-5-tetrazolio]-1,3-benzene sulfonate) colorimetric assay (Sigma-Aldrich, St. Louis, MO, USA) was conducted. HGFs were seeded in sterile 96-well plates (Greiner Bio-One, Frickenhausen, Austria) at a density of 10^4^ cells/well and were allowed to attach for 24 h in the humidified incubator at 37 °C. After 24 h incubation, the culture medium was replaced by a given eluate (24 h, 48 h, 72 h, 10 days, 30 days, and 3 months) and incubated for an additional 24 h. The viability of HGF-1 cells in a specific eluate was assessed the next day, following a 2 h incubation at 37 °C subsequent to the addition of 10 µL of WST-1 reagent to each well. Using a microplate spectrophotometer (Varioskan, Thermo Fisher Scientific, Waltham, MA, USA) and SKanIT RE for Varioskan software (Ver.2.2, Thermo Fisher Scientific), optical densities were measured according to the manufacturer’s standard protocol at 440 nm and a reference absorbance at 600 nm. Following the subtraction of the blank control, the absorbance values at 600 nm were deducted from those obtained at 440 nm to adjust for plate background variations. Cell viability was then calculated as a percentage relative to the negative control (untreated cells) as represented in Equation (1):

(1) $$\%Viability=100\times \left(A440-\left.A600\right)\right.treated/\left(A440-\left.A600\right)control\right.$$

Cellular viability of 100% was attributed to the HGFs grown in the control cell culture medium. Samples of each material at a given time point were tested in triplicates and all assays were repeated as independent experiments at least twice. The results were interpreted according to ISO 10993-5 [30].

### 2.8. Statistical Analysis

The Kolmogorov–Smirnov and Levene’s tests were employed to determine the test results’ normal distribution and equality of variances. A one-way analysis of variance (ANOVA) with Tukey post hoc test was employed for the cell cytotoxicity data. Additionally, Welch’s ANOVA with Games–Howell post hoc test was used in the event of variance heterogeneity. For the SBS, adhesive layer thickness, and the RTPN results, multiple independent Student’s *t*-tests (α = 0.05) were employed. The ARI score was evaluated using the Fisher–Freeman–Halton exact test, a non-parametric alternative to the Chi-squared test for multi-category contingency tables, followed by Z-tests for column proportion comparisons with Bonferroni-adjusted p values. Results were considered statistically significant for *p* ≤ 0.05 (95% confidence level). The statistical analysis was carried out using SPSS computer software (IBM SPSS Statistics Version 27, International Business Machines Corporation, NY, USA). The partial eta-squared statistic showed the practical relevance of each term based on the ratio of the variation attributed to the effect. Additionally, a Weibull analysis was employed to evaluate the bond strength data. The Weibull model is used to describe the probability of failure in brittle materials as a function of applied stress. In this study, it was applied to model the cumulative probability of failure $P_{f}$ at a certain stress level $\sigma_{c}$ and is expressed in Equation (2).

(2) $$P_{f}\left(\sigma_{c}\right)=1-exp\left[{-\left(\sigma_{c}-\left.\sigma_{0}\right)\right.}^{m}\right]$$

In this equation, $P_{f}$ is the failure probability $\sigma_{c}$ is the measured strength, $\sigma_{0}$ is the characteristic strength at 63% failure probability, and m reflects strength variability. When the expression is converted into its double logarithmic form, a linear relationship is obtained (Equation (3)). The slope of this line represents the Weibull modulus m, and the x-intercept corresponds to the logarithm of the characteristic strength $\sigma_{0}$.

(3) $$\ln\ln\left\lfloor 1/(1-P_{f}\left(\sigma_{c}\right))\right\rfloor =m\ln\left(\sigma_{c}\right)-m\ln\left(\sigma_{0}\right)$$

## 3. Results

### SBS Test

**Figure 2:**
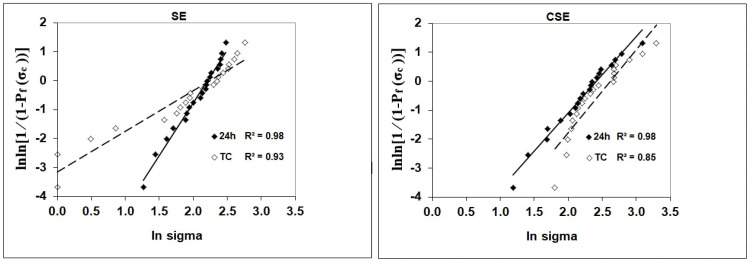
Weibull distribution as a function of material and storage.

The SBS and Weibull statistics of the adhesive groups and aging conditions are presented in Table 2. Multiple independent Student’s *t*-tests showed significant differences between the two adhesives and within the TC of each adhesive group for the SBS variable. No statistically significant differences were observed between the adhesive groups at 24 h water storage. After TC, CSE exhibited significantly better results than SE (*p* = 0.014). No decrease in SBS after TC could be observed in the adhesive groups. SE achieved the highest Weibull modulus at 24 h (Figure 2 and Table 2). Although the control group CSE had a lower Weibull modulus at 24 h than the experimental group, TC did not affect the Weibull modulus for this group. In contrast, TC led to a decrease in the Weibull modulus for the experimental group.

**Figure 3:**
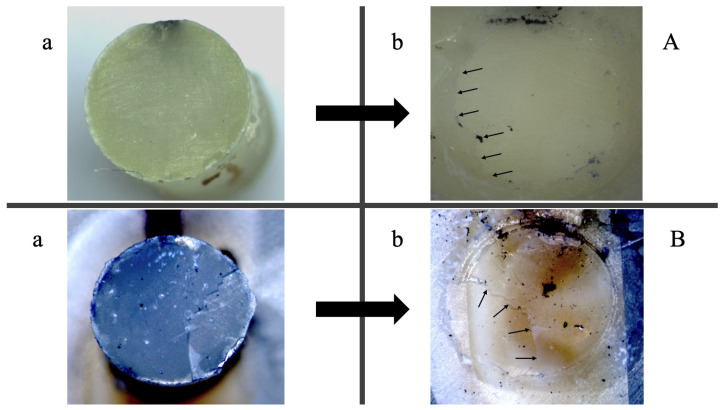
Fractographic analysis using light microscopy of the same sample following shear bond strength testing after 24 h. (**A**) SE; (**B**) CSE. Images (a) illustrate the composite fractured surface, while images (b) depict the corresponding dentin fracture surface. Small arrows indicate adhesive remnants on dentin surfaces, exemplifying adhesive fractures in both cases. The reference adhesive (CSE) demonstrated superior adhesion to dentin, whereas the experimental adhesive (SE) adhered more effectively to the composite.

Results of the fractographic analysis showed that all fractures occurred adhesively. A representative example of the adhesive fracture mode for both adhesives, SE and CSE, is illustrated in Figure 3.

The Fisher–Freeman–Halton exact test showed a significant dependency (*p* < 0.001) between the adhesive used and the mARI score in both 24 h and TC groups. Table 3 shows the distribution count and percentage of each adhesive group in correlation with the mARI score.

**Figure 4:**
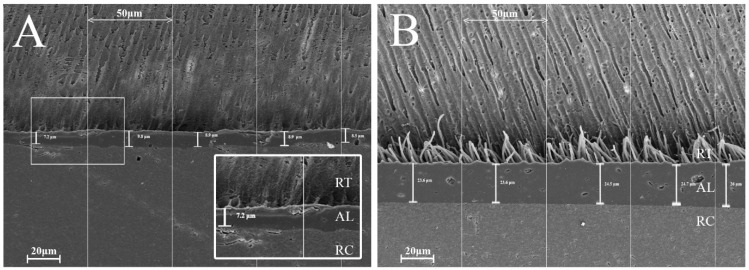
SEM images of the adhesive interfaces: (**A**) group SE, (**B**) group CSE. RT = resin tags, AL = adhesive layer, RC = resin composite. The images highlight differences in resin tag formation, quantity, and depth of penetration into dentinal tubules between the two adhesive systems. Variations in adhesive layer thickness are also observable.

Multiple independent Student’s *t*-tests showed significant differences between the two adhesives regarding the variables adhesive layer thickness and RTPN (Table 4). In comparison, no statistically significant differences could be observed in terms of adhesive layer thickness between the two adhesive groups. In the case of the RTPN, the results showed statistically better results for the CSE adhesive group (*p* < 0.001). SE produced a uniform adhesive layer comparable with the control group CSE on the studied SEM images. On the other hand, SE showed a very scarce presence of resin tags compared to CSE. A hybrid layer was difficult to identify along the length of the interface of the specimens. Figure 4 shows SEM images with the particularities of each adhesive group.

Regarding cellular cytotoxicity, the effects of the parameters “adhesive”, “time”, and the interaction between “time and adhesive” were studied. Among them, the following effects were statistically significant: “time” (*p* < 0.001, η_P_^2^ = 0.316) and the interaction between “time and adhesive” (*p* < 0.001, η_P_^2^ = 0.242).

**Figure 5:**
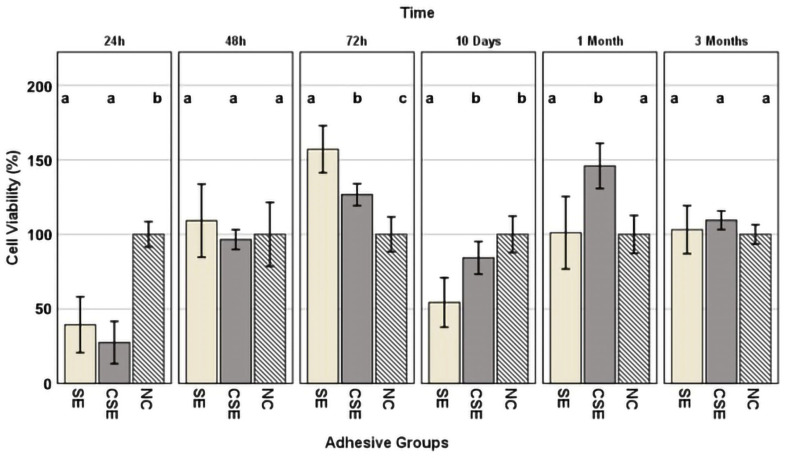
Cell viability values ± standard deviation, expressed as a percentage of the negative control (NC), presented by time frame. Superscript letters indicate statistically significant differences between the NC and adhesive groups. Columns sharing the same letter are not significantly different. Statistical analysis was performed using Tukey’s HSD post hoc test and, in cases of variance heterogeneity, the Games–Howell test (α = 0.05).

Figure 5 shows the percentage (%) of cell viability of the HGF-1 cells exposed to eluates from the two adhesive groups at different time frames, including the negative control NC. This figure also shows statistically significant differences between each adhesive group within a single time frame. After the first 24 h of elution, both adhesive groups showed reduced cellular viability when compared to the negative control NC: SE (39%, *p* < 0.001) and CSE (27%, *p* < 0.001). Following the next time frame (48 h of elution), no statistically significant differences could be seen between the adhesive groups and the negative control. While at 72 h of elution, all groups differed statistically from one another, no trace of reduced cellular viability could be highlighted (viability ≥ 100%). After 10 days of elution, the lowest cellular viability was shown in the SE group (54%), differentiating itself statistically from both the control group CSE (83%, *p* = 0.008) and the negative control NC (*p* = 0.004). Similar to the 72 h eluates, after one month of elution, although no traces of reduced cellular viability could be seen, CSE was statistically different from SE (*p* = 0.011) and NC (*p* = 0.001). The last time frame (three months of elution) showed no differences between the adhesive groups and the NC.

**Figure 6:**
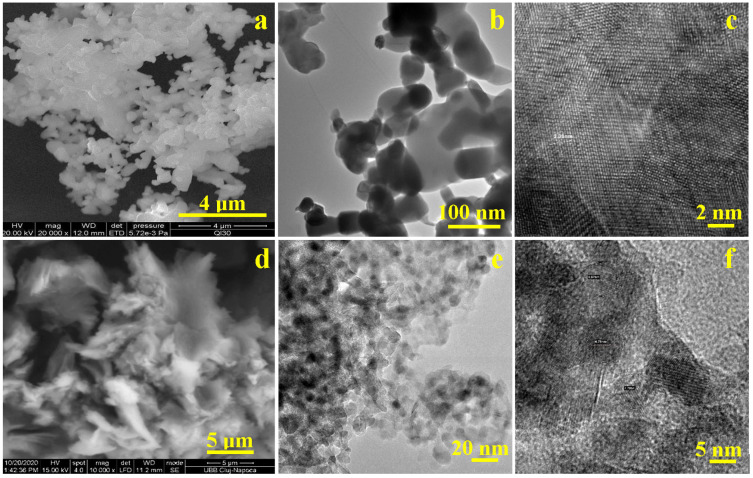
Micro- and nano-structural characterisation of the synthesised HA-ZnO mixture: (**a**) SEM image of HA and ZnO nanoparticle, (**b**) bright-field TEM (BFTEM) of HA-ZnO aggregates, (**c**) HRTEM image of HA-ZnO: (**d**) SEM image of graphene oxide (GO), (**e**) bright-field TEM (BFTEM) of GO-ZrO_2_ fillers, (**f**) HRTEM image of GO-ZrO_2_ fillers.

The morphological characteristics of the synthesised fillers analysed by SEM and bright-field transmission electron microscopy (H-7650 120 kV Automatic TEM, Hitachi, Japan) are presented in Figure 6. SEM images identified a dense and homogeneous mixture of HA and ZnO nanoparticles with a strong coalescence tendency, making the nanofeatures difficult to visualize. These were visible in bright-field transmission electron microscopy (BFTEM) and revealed a cluster of nanoparticles, indicating aggregated or densely packed structures typical of HA-ZnO composites (Figure 6b). The elongated HA nanoparticles were clearly visible and had a length of approximately 80–110 nm and a diameter of approximately 50 nm. These were very well mixed with ZnO nanoparticles, which had rounded shapes and sizes of approximately 40–60 nm. The HRTEM in Figure 6c shows a strong connection between the crystal planes in HA and ZnO, indicating some interaction between the detected nanocomponents, which enhanced a synergistic effect within the composite. The presence of such ultrastructural particles indicated a high surface area, a property advantageous for biomedical and photocatalytic applications. The small particles obtained in this synthesis may be due to an increased number of crystallization centres (SEM image). This may have been caused by the addition of a factor based on a mathematical equation to the reaction mixture of Ca(OH)_2_ and H_3_PO_4_. This likely led to the formation of more crystallization centres and thus to smaller crystal sizes.

Figure 6d shows a scanning electron microscopy (SEM) image of graphene oxide (GO). The characteristic wavy morphology of graphene oxide (GO) indicated a sheet-like structure. Zirconia nanoparticles intercalated into the GO influenced the nanostructure, leading to the formation of ultrathin sheets with a wavy and crumpled texture, in which rounded ZrO_2_ nanoparticles with a rounded shape were embedded (Figure 6e). The observed nanostructure of the synthesized GO-ZrO_2_ nanocomposite showed a random distribution of the ZrO_2_ nanoparticles across the surface of the GO sheets. This uniform distribution was likely the result of heterogeneous nucleation and strong interfacial interactions between the ZrO_2_ nanoparticles and the GO substrate. The HRTEM image of the GO-ZrO_2_ composite in Figure 6c shows clearly defined lattice fringes corresponding to the (101) plane of ZrO_2_, confirming the crystalline nature of the zirconia phase. However, due to the ultrathin nature of reduced graphene oxide (rGO), its own lattice fringes were not visible by HRTEM.

## 4. Discussion

This study outlined the successful synthesis of functionalized GO and HA nano-powders, which were subsequently incorporated into an experimental two-step, self-etch dental adhesive formulation. The synthesized GO-ZrO_2_ and HA-Zn powders were selected based on various favourable attributes documented in the literature, particularly concerning the mechanical reinforcement of the polymeric structure [15,31], enhanced durability of the adhesive–dentin interface [8], remineralisation of the tooth substrate [32], bioactivity and antibacterial properties [14]. Recent reports further indicate that incorporating just 0.1–5% graphene by weight as a nanofiller in RBCs or epoxy composites can consistently enhance the mechanical properties of RBCs [31,33]. Another major advantage of GO is its antibacterial properties. Although it is difficult to pinpoint the exact antimicrobial mechanism of graphene due to its diverse intrinsic properties, especially when combined with other substances, it is known that graphene interacts with microorganisms in several ways: it can penetrate and damage cell membranes [14], wrap around cells leading to mechanical stress, extract phospholipids through chemical interactions with the bacterial lipid layers due to its hydrophobicity, produce reactive oxygen species (ROSs) that cause oxidative stress and cellular damage and pull electrons from microbial membranes, destabilizing them [34]. GO has also been shown to prevent bacterial attachment, particularly in the case of *Streptococcus mutans* [35]. In addition to the described antibacterial properties and the ability to reinforce polymer matrices, graphene oxide (GO) may also enhance the remineralisation potential of adhesive systems. Even small concentrations of GO incorporated into dentin adhesives have been reported to promote mineral deposition at the adhesive–dentin interface. This effect is attributed to GO’s hydrophilic character, which facilitates calcium ion attraction and promotes HA nucleation [8].

To enhance the osteoconductivity, biocompatibility, and remineralisation potential of GO, HA was incorporated into our adhesive formulation. HA is widely recognized for its excellent biocompatibility and bioactivity, making it a valuable addition to dental restorative materials. Studies have demonstrated its efficacy in remineralising early carious lesions and reducing hybrid layer degradation, primarily due to its ability to resist enzymatic activity and interact favourably with dentin [21,36,37]. Despite the inherent brittleness and low fracture toughness of HA, its hexagonal crystal structure allows the incorporation of metal ions such as Zn, which not only improves its mechanical properties but also confers significant biological functionalities [25].

Moreover, Zn and ZnO exhibit strong antibacterial activity against both Gram-positive and Gram-negative bacteria, including *Streptococcus mutans*. In this context, it has been described that ZnO nanoparticles utilize different primary antibacterial mechanisms. They are able to generate reactive oxygen species (ROSs) that disrupt bacterial membranes and DNA, physically penetrating cells via electrostatic interactions and releasing Zn ions to inhibit bacterial growth and promote cell death [38]. Additionally, Zn inhibits matrix metalloproteinases (MMPs) by binding to collagen cleavage sites, thus protecting dentin collagen from enzymatic degradation and collapse [39]. These combined properties of HA and Zn highlight their synergistic potential in enhancing the performance and longevity of dental adhesives.

With a view to a mild self-etch approach we added PAA in the composition of the primer. Often used in the composition of glass ionomers, PAA is sufficiently acidic to remove the smear layer without excessively demineralizing the dentinal surface. The significance lies in the fact that the resulting adhesive only interacts superficially with the dentin/enamel and does not measurably dissolve HA crystals but rather holds them in place. HA not only encapsulates and protects the collagen fibrils but also provides a substrate for the chemical interaction of the adhesive, achieving a strong and stable bond to dentin [3,40]. This is in line with a study by Gonçalves et al., in which 25% PAA was used as a mild etchant prior to applying a universal adhesive. The effect was evaluated using a Fourier transform infrared spectroscopy (FTIR) analysis, indicating that PAA preserved more phosphate and carbonate groups in the dentin structure, indicating less aggressive demineralization. This preservation allowed more HA to remain on the collagen surface, facilitating chemical interactions with the adhesive monomers. The reduced exposure of collagen minimized enzymatic degradation, supporting long-term bond durability [40].

The presence of water is crucial in self-etch adhesive systems as it ionizes the acidic monomer, enabling it to etch and infiltrate tooth substrates simultaneously [41]. Another key step was to identify a hydrophilic polymer compatible with PAA in an aqueous solution. HEMA fitted this requirement. As a low molecular weight, hydrophilic monomer, HEMA acts as a solvent, enhancing the penetration of dentinal substrates, and it is able to polymerize with the other monomers contained in the adhesive formulation.

To achieve precise control over each component, we synthesized bis-GMA oligomers as the polymer matrix for the experimental adhesive along with the previously described synthesized powders. bis-GMA is commonly used in dental adhesives and composite resins due to its high molecular weight, reduced polymerization shrinkage, and rapid curing properties [42]. However, these properties also result in increased viscosity, rigidity, and a lower monomer conversion rate [43]. To address these limitations, TEGDMA/HEMA, which are often incorporated as diluents, were added to the formulation to reduce viscosity and improve the flexibility of the resulting polymer [42,44]. As the experimental self-etch adhesive is a prospective material for clinical use, its performance was compared to the current gold standard CSE. CSE’s mild self-etch formulation contains the functional monomer 10-MDP, which guarantees a stable dentin bond duration and is therefore recommended by van Meerbeek et al. [3].

Based on our recent findings reported in a previous paper, we selected a filler concentration of 0.2% GO-ZrO_2_ by weight for our experimental setup, as it demonstrated superior bond strength even after TC, compared to an adhesive with a higher graphene concentration of 5% by weight [45]. Additionally, a recent study by Bregnocchi et al. investigated various concentrations of graphene nanoplatelets (0.1% by weight, 0.2% by weight, and 0.5% by weight) which were incorporated in a commercial etch-and-rinse adhesive. Their results indicated that incorporating 0.2% graphene nanoplatelets by weight yielded the highest bond strength, exhibited the strongest antibacterial activity against *Streptococcus mutans*, and resulted in a uniform particle distribution within the adhesive without agglomerations, while also maintaining rheological properties similar to those of the control [46]. Moreover, previous studies limiting GO content in RBCs for aesthetic purposes further motivated our choice of a 0.2% GO-ZrO_2_ filler in our experimental setup [47,48].

Our mild self-etch approach in adhesive design combined with the advantages of the synthesized powders described above, yielded an adhesive with bond strength performance comparable to that of CSE, even after TC. Although the shear bond strength data did not reveal a clear trend, the decrease in the Weibull modulus in the experimental group indicated that the adhesive was only slightly impacted by hydrolytic stress during TC. In contrast, the control group exhibited less scatter among individual measurements, suggesting more consistent and reliable bond strength data after TC. Interestingly, while the experimental adhesive showed comparable shear bond strength to the control at 24 h, it demonstrated superior performance in terms of reliability at that stage, as indicated by its higher Weibull modulus (see Table 2). This difference implies that the experimental adhesive was more susceptible to degradation under accelerated aging conditions than the control adhesive.

In etch-and-rinse adhesives, bond degradation is frequently associated with insufficient resin infiltration into demineralized dentin at the base of the hybrid layer, creating a potential site for interfacial failure [49,50]. In contrast, two-step self-etch adhesives such as those utilized in this study are less likely to leave areas of inadequately infiltrated demineralized dentin. Self-etch adhesives use acidic monomers to simultaneously demineralize and infiltrate dentin [3], though some studies have observed a mismatch between the depth of demineralization and resin infiltration indicating that unprotected demineralized dentin may still persist as a vulnerable area [51]. These exposed or partially coated collagen fibrils are more susceptible to hydrolysis, enzymatic degradation, and thermal stress, which can accelerate bond deterioration over time [52]. The performance of the experimental adhesive, which combines PAA, bis-GMA oligomers, TEGDMA, and HEMA, reflects these challenges, particularly in the context of TC. The hydrophilic components of the adhesive, such as HEMA and PAA, are well documented for their tendency to absorb water, which can exacerbate hydrolytic degradation and lead to localized microstructural changes within the polymer network [53]. Despite this, the overall bond strength remained stable. However, the reduction in the Weibull modulus after TC suggested increased variability in bond performance across specimens, likely due to uneven water uptake and swelling [54]. This behaviour is characteristic of adhesives containing hydrophilic monomers, as water sorption can weaken localized areas without uniformly compromising the adhesive layer [7].

The reliability and the long-term bond stability of CSE can be attributed to its inclusion of 10-MDP. This functional monomer forms stable ionic bonds with calcium in the residual HA of the hybrid layer, which minimizes decalcification and promotes the formation of a poorly soluble calcium salt, supporting sustained bond strength with dentin over time. Additionally, 10-MDP interacts with collagen using its hydrophobic groups to enhance adhesion to dentin and resist degradation. By encapsulating collagen within HA, the mild self-etch approach helps maintain the collagen structure and prevents collapse [3]. The durability of this bond was validated in clinical settings, as demonstrated in a 13-year randomized controlled clinical trial conducted by Peumans et al. [55]. This study reported a 96% success rate for CSE in treating non-carious class V lesions, underscoring the clinical efficacy and reliability of this bonding approach over an extended period.

One of the most remarkable properties of 10-MDP is its ability to form a self-assembled nano-layered structure at the adhesive interface. These alternating layers of MDP–calcium salts create a robust and chemically stable interface with HA, offering resistance to acid, enzymatic degradation, and hydrolytic stress. This nano-layering mechanism not only enhances the mechanical strength of the adhesive interface but also acts as a moisture barrier, preventing water ingress and maintaining the integrity of the hybrid layer over time [56]. In contrast, adhesives lacking 10-MDP, such as the experimental adhesive, cannot form these chemically bonded nano-layered structures. Without this protective interface, the adhesive relies primarily on micromechanical retention, making it more vulnerable to hydrolytic and thermal stress during aging processes like thermocycling [3]. 10-MDP was deliberately not introduced in the experimental adhesive or primer to avoid overlapping with potential improvements that could be attributed to the new chemical composition. This decision provided a clearer understanding of how the experimental adhesive compared to CSE. The fact that bond strength values remained stable following 24 h immersion and TC and were comparable to those of the control demonstrated the remineralisation capabilities and the mechanical reinforcement potential provided by the added particles (HA, GO, Zr). Our findings align with those of Yasser F. AlFawaz et al., who demonstrated that integrating 2% HA-GO by weight in an etch-and-rinse adhesive achieved the highest microtensile bond strength under both TC and normal conditions. Energy-dispersive x-ray spectroscopy in their study also showed traces of Ca and P in their HA/GO 0.5% by weight group, which are crucial components in the remineralisation potential of hard dental tissue [21].

The exclusively adhesive mode of fracture confirmed that the measured data accurately reflected bond strength. A further analysis of the fracture sites using the mARI score showed that SE adhered better to the RBC both after 24 h immersion and TC, whereas CSE adhered in both cases better to dentin. This result indicates that CSE demonstrated superior adhesion to dentin, likely due to the ability of the contained monomer 10-MDP to form stable, self-assembled nano-layers through ionic bonding with Ca in dentine’s HA as mentioned earlier [56]. These nano-layers acted like self-organized filler particles that reinforced the adhesive layer whereas PAA did not. Moreover, the filler particles could have enhanced the bulk properties of the adhesive rather than specially reinforcing the dentin–adhesive interface. To further investigate the dentin–adhesive interface, SEM examinations were performed. Both adhesive groups exhibited similar adhesive layer thicknesses. However, the control group outperformed the experimental group in terms of resin tag penetration (RTPN). In the experimental group, the adhesive’s penetration potential into the dentinal tubules was visibly limited with only the sparse presence of short, needle-like resin tags. Additionally, no cone-shaped tags or lateral branches were noted in our specimens, unlike the structures described by Ferrari and Davidson, who suggested that such features could positively influence shear bond strength (SBS) values [57]. This limited penetration may be explained by the mild self-etch approach employed. During substrate penetration, the acidic monomers in the adhesive are gradually buffered by the mineral content in the dentin, reducing their capacity to etch the substrate further. Additionally, factors such as the incomplete dissolution of smear plugs, particle agglomeration within the adhesive, orientation of dentinal tubules, and irregularities in the smear layer may hinder effective adhesive infiltration into the tubules [58]. However, there is no conclusive evidence that either adhesive layer thickness or the quantity of resin tags directly enhances the mechanical properties of the adhesive, which is in agreement with our data [57]. Our findings also suggest that the adhesion relied more on chemical interaction due to particle reinforcement than on purely micromechanical bonding.

As mentioned earlier, the antibacterial activity of GO is remarkable, but there is concern about cytotoxicity. When a compound is active against microbes, it is likely to be active in other body cells [59]. The ideal biologic methodology for testing the biocompatibility of dental adhesives would consist of in vivo experiments. Although in vitro studies are limited due to the absence of biological and physiologic components that are impossible to reproduce entirely in vitro, they are suitable for determining whether a material contains significant quantities of biologically harmful components. In the present study, we assessed the cytotoxicity and the biocompatibility of the adhesives using the WST-1 colorimetric assay in six different time frames (24 h, 48 h, 72 h, 10 days, 30 days, and 3 months). We used the HGF-1 cell line for the colorimetric assay as these cells come into close contact with resin-based dental materials and are frequently used in cell viability assays [60]. Results were interpreted according to ISO 10993-5, which states that a medical material has cytotoxic potential if the viability is reduced by more than 70% compared to the negative control [30]. Analysing the graph shown in Figure 5, the 24 h eluate of both experimental and control adhesives showed cytotoxic potential (SE: 39% viability and CSE: 27% viability). The 72 h eluate of SE showed viability measurements surpassing 150% which were considered non-cytotoxic but rather were potentially cell-growth-inducing. The 10-day eluate of SE (54% viability) again showed cytotoxic potential. It should be noted that the 10-day eluates represented cumulative extracts, including materials released from day 3 (72 h) to day 10. A neutralization of the cytotoxic effects was evident in both adhesive groups over a longer time frame of up to 3 months.

In general, the toxicity of dimethacrylate polymer networks is mainly caused by leaching low molecular weight substances, which can induce harmful effects on oral tissue. Low molecular substance release is either caused by incomplete polymerization/conversion or by chemical degradation of the dimethacrylate polymer network [61]. This also applies to resin-based dental adhesives, where the best-known leachable components responsible for cytotoxic effects on oral tissues are unreacted dimethacrylate monomers. Additionally, other substances such as photoinitiators and catalysts could be leached from the adhesive structure and induce cytotoxic effects on oral tissue. The concentration and the combination of different resinous monomers play a significant part in their potential role in causing cell damage [62,63]. The following monomers have been ranked by decreasing toxicity in the following order: HEMA > TEGDMA > UDMA > bis-GMA [64]. Despite the lower toxicity compared to other methacrylates as presented above, when uncured, reports on bis-GMA show that it can cause cytotoxicity and genotoxicity in various mammalian cell types such as gingival fibroblasts [64,65], lymphocytes [66], and dental pulp cells [67]. Also, bis-GMA has been shown to stimulate ROSs and induce cell cycle arrest and apoptosis in dental pulp cells [67].

As described in Section 2.1, we utilized self-synthesized bis-GMA oligomers to enhance both the mechanical properties of the adhesive and address the cytotoxicity concerns associated with unreacted bis-GMA monomers. Due to their larger molecular size and reduced mobility, oligomers exhibit a significantly lower potential for elution, minimizing the release of harmful substances into surrounding tissues. This not only improved biocompatibility but also maintained the adhesive’s performance. Furthermore, the controlled synthesis of bis-GMA oligomers ensured their stable integration into the polymer matrix, reducing cytotoxic effects even further. Studies have shown that the oligomerization of bis-GMA effectively reduces the leaching of toxic components without compromising its mechanical properties [17,68]. In addition, both HEMA and TEGDMA, included as dilution monomers in our experimental adhesive, have been reported to influence cell viability by inducing reactive oxygen species (ROS) production and depleting glutathione (GSH) levels in human dental pulp cells [69,70]. These findings suggest that the observed cytotoxicity may stem from the combined effects of residual monomers and photoinitiators, which highlights the importance of optimizing polymerization processes and monomer selection to enhance biocompatibility [71]. Thus, we conclude that the experimental adhesive possibly had cytotoxic potential at two time frames, 24 h and 10 days; however, it showed remarkable results in the other time frames especially in the 72 h eluate, which reveals that GO is not only non-toxic to HGF-1 cells but can also increase their proliferation activity. Similar phenomena have been observed in other studies, involving various cell types [72,73,74,75,76,77]. Integrated into collagen membranes, GO led to the proliferation of HGF-1 cells and mediation of inflammatory events even at low concentration. One study reported enhanced proliferation and mitochondrial activity by L929 fibroblasts when cytotoxic tests were conducted using pristine graphene [75]. Graphene has been observed to enhance the proliferation of human mesenchymal stem cells and facilitate their differentiation into dental tissue such as osteoblasts [73]. GO integration into the experimental dental adhesive SE positively affected the viability of HGF-1 cells, producing comparable results with those of CSE. Multiple studies have shown that graphene’s cytotoxicity is influenced by various factors such as concentration, shape, size conformation, dispersibility, and particle functionalization [48,78]. These complexities make it challenging to determine its precise toxicity levels. Nonetheless, graphene’s potential for biocompatibility deserves closer attention.

This study does have limitations that warrant consideration. One primary drawback of incorporating graphene in higher amounts into adhesive systems is its dark colour, which poses aesthetic challenges, an increasingly important factor in restorative dentistry. The concentration used in this study was carefully optimized to minimize such concerns. Furthermore, as an in vitro study, these findings should be applied to clinical practice with caution, as in vitro results may not fully translate to in vivo conditions.

## 5. Conclusions

The incorporation of functionalized GO and HA nano-powders facilitated the development of a novel self-etch dental adhesive with enhanced performance characteristics. The experimental adhesive demonstrated comparable biocompatibility to the gold standard CSE adhesive with evidence of improved cellular viability during specific aging intervals. Bond strength to dentin remained stable and reliable in the early phases, while TC exhibited only a minor impact on its long-term performance. These findings underscore the potential of GO and HA-functionalized adhesives as innovative candidates for clinical applications in restorative dentistry.

## Figures and Tables

**Table 1:** Chemical composition of the materials used, indicated in weight %.

Code	Organic Matrix	PAA	Ethanol	Inorganic Filler [wt.%]	Lot No.
SE (bond)	bis-GMA _(0-2)_ oligomers [35%] TEGDMA [13%] HEMA [40%]	5	-	GO-ZrO_2_ [0.2%] HA-Zn [6.8%]	-
SE (primer)	HEMA [50%]	35	15	-	-
CSE (bond)	MDP HEMA DM bis-GMA	n.s.	n.s.	SiO_2_ [n.s %]	CF 0039
CSE (primer)	MDP HEMA DM	n.s	n.s	n.s	CF 0039
AF	ormocer			Ba–Al–Si-glass, SiO_2_ [84.0%]	1,537,600

Abbreviations: SE = self-etch experimental adhesive; CSE = Clearfill SE BOND 2, Kuraray Co., Ltd., Kurashiki, Japan; bis-GMA _(0–2)_ oligomers = (2,2-bis[4-(2-hydroxy-3-methacryloxypropoxy) phenyl]-propane), synthetized at UBB; TEGDMA = triethylene glycol dimethacrylate; HEMA = 2-hydroxyethylmethacrylate, MDP = 10-methacryloyloxydecyl dihydrogen phosphate; DM = dimethacrylate; ormocer = organically modified ceramic; PAA = polyacrylic acid, GO-ZrO_2_ = (graphene oxide–zirconia, synthetized at UBB), HA-Zn = (hydroxyapatite–zinc, synthetized at UBB), SiO_2_ = silanated colloidal silica, n.s. = not specified.

**Table 2:** Mean values ± standard deviation for shear bond strength (MPa) and Weibull modulus with associated confidence intervals. Lowercase superscript letters indicate significant differences between materials under the same storage condition, while uppercase letters mark significant differences within the same material before and after TC. Statistical comparisons were performed using independent Student’s *t*-tests with a significance level of α = 0.05.

Adhesive	Shear Bond Strength (SBS) (MPa)		Weibull Modulus (m)	
	**24 h**	**TC**	**24 h**	**TC**
SE	8.2 ± 2.4 ^a A^	8.2 ± 4.6 ^a A^	3.7 [3.4–3.8]	1.4 [1.2–1.5]
CSE	10.0 ± 3.2 ^a A^	12.2 ± 5.2 ^b A^	2.6 [2.5–2.8]	2.9 [2.3–3.5]

**Table 3:** Count and percentage of specimens receiving each mARI score, grouped by adhesive type and storage condition (24 h and TC). The mARI (modified Adhesive Remnant Index) was determined using scores 1 (0–25%), 2 (25–50%), 3 (50–75%), and 4 (75–100%) for the adhesive film remaining on the dentin surface. Statistical comparisons between adhesive groups for each score were performed using the Fisher–Freeman–Halton exact test. Lowercase letters indicate significant differences, based on Z-tests with Bonferroni-adjusted *p*-values.

24 h				TC			
mARI Index		SE	CSE	mARI Index		SE	CSE
1	Count	17 ^a^	0 ^b^	1	Count	20 ^a^	1 ^b^
	%	85%	0%		%	100%	5%
2	Count	1 ^a^	3 ^a^	2	Count	0 ^a^	5 ^b^
	%	5%	15%		%	0%	25%
3	Count	1 ^a^	3 ^a^	3	Count	0 ^a^	3 ^a^
	%	5%	15%		%	0%	15%
4	Count	1 ^a^	14 ^b^	4	Count	0 ^a^	11 ^b^
	%	0%	70%		%	0%	55%

**Table 4:** Mean values ± SD for the RTPN (number of resin tags/50 μm) and adhesive layer thickness (μm). Different superscript letters indicate statistically significant differences between the two materials. Independent Student’s *t*-test (α = 0.05).

Adhesive	Adhesive Layer Thickness (μm)	RTPN
SE	5.4 ± 3.4 ^a^	1.6 ± 2.1 ^a^
CSE	8.1 ± 9.9 ^a^	24.4 ± 5.7 ^b^

## Data Availability

The raw data supporting the conclusions of this article will be made available by the authors on request.
